# Risperidone for the Management of Treatment-Resistant Anxiety in a Patient with Ehlers-Danlos Syndrome: A Case Report

**DOI:** 10.7759/cureus.9493

**Published:** 2020-07-31

**Authors:** Katherine Niedt, Cristina Young, Nidhi Sharoha

**Affiliations:** 1 Psychiatry, Stony Brook University Hospital, Stony Brook, USA; 2 Medicine, Renaissance School of Medicine at Stony Brook, Stony Brook, USA; 3 Psychiatry and Behavioral Sciences, Stony Brook University Hospital, Stony Brook, USA

**Keywords:** clinical anxiety, ehlers danlos syndrome, risperidone

## Abstract

Patients with Ehlers-Danlos syndrome (EDS) have a higher prevalence of comorbid anxiety disorders. Due to the complex nature of these patients, there is often a delay in the diagnosis of these disorders as well as insufficient management of their anxiety symptoms. Current treatment options are often insufficient or poorly tolerated by patients, creating barriers to proper treatment. We hypothesized that patients with EDS and comorbid anxiety, who have failed multiple medication trials, may benefit from a trial of risperidone. In this case report, we discuss the successful management of treatment-resistant anxiety in a patient with EDS with the use of risperidone. Management of comorbid anxiety in these patients is essential, as untreated anxiety can result in increased somatic sensation sensitivity and poor social functioning. Once underlying anxiety disorders are addressed, patients with EDS can better cope with chronic pain symptoms and are more likely to build a therapeutic alliance with their treating physicians. This results in an improved prognosis, social functioning, and overall quality of life.

## Introduction

Ehlers-Danlos syndrome (EDS) refers to a hereditary group of disorders that affect connective tissue matrix proteins. Characteristics of EDS include joint hypermobility, skin hyperextensibility, and tissue fragility [1]. EDS is a multisystemic disease, affecting the musculoskeletal system, cardiovascular, gastrointestinal, mucocutaneous, urogynecological, ocular, and neuropsychiatric systems. Neuropsychiatric manifestations include proprioception dysfunction, dysautonomia, and anxiety [1].

Literature has demonstrated that there is a strong association between EDS and comorbid psychiatric disorders. Some examples of these disorders include anxiety, depression, eating disorders, and neuro-developmental disorders [2]. In a systematic, retrospective chart review of 391 patients, 49.4% were reported to have a psychiatric disorder, with 28.9% having two or more disorders, and 26.6% having an anxiety disorder [3]. These patients often also suffer from chronic pain, which is associated with an increased incidence of comorbid psychiatric disorders [3]. For example, one study found that 29% of patients with chronic pain also suffer from depression [4]. Furthermore, in rheumatologic patients with joint hypermobility syndrome, also known as Ehlers-Danlos III, 70% of patients were found to have some kind of anxiety disorder earlier in life [4]. Overall, these patients were ten times more likely to suffer from anxiety than patients without joint hypermobility [4]. 

Anxiety disorders in patients with EDS and joint hypermobility syndrome often go unrecognized, and despite the high prevalence of anxiety disorders in patients with EDS, less than 25% of patients receive treatment [4]. Current treatments for anxiety disorders in these patients include cognitive-behavioral therapy (CBT) for the management of comorbid chronic pain, as well as pharmacologic treatments [4]. Pharmacological therapies include selective serotonin reuptake inhibitors (SSRIs), which are first line, tricyclic antidepressants (TCAs), such as imipramine and clomipramine, monoamine oxidase inhibitors (MAOIs), benzodiazepines, and beta-blockers [5]. Each of these medications has the potential for drug-drug interactions.

A prior case report discussed the management of anxiety, depression, and borderline personality disorder in a 25-year-old female with EDS who was treated with sertraline 150 mg qd (once a day), gabapentin 600 mg BID (twice a day), and lithium 300 mg BID (to help augment SSRI and attempt to decrease suicidality). However, this patient’s anxiety symptoms persisted despite the use of medications [6]. A large number of psychiatric medication regimens have been employed for the management of anxiety in patients with EDS, however they are often unsuccessful or poorly tolerated [6]. This requires providers to better tailor therapies to each individual in order to sufficiently treat patients with treatment-resistant anxiety disorders. Our hypothesis is that patients with EDS and comorbid anxiety disorders, who have had failed multiple medication trials, may benefit from a trial of risperidone. Knowledge about the benefits of this intervention can be very helpful for providers as patients with EDS are often repeatedly hospitalized and anxiety management can become challenging due to several medical comorbidities associated with EDS. In the case report below, we discuss the successful use of risperidone in a young female diagnosed with EDS and treatment-resistant generalized anxiety disorder.

## Case presentation

Our patient is a 34-year-old Caucasian female, with a past psychiatric history of unspecified depression and anxiety, and a complex medical history most notable for EDS, complicated by chronic pain (and longstanding history of opioid use disorder), prior motor vehicle accident with hardware in legs and back, dysautonomia including postural tachycardia syndrome, gastroparesis with gastrostomy tube and jejunostomy tube, non-epileptiform seizures, hypothyroidism, and chronic migraines, who was admitted to the hospital’s medical service for opioid withdrawal. One month prior to this hospitalization, patient had been hospitalized for decreased oral intake, and at this time, her pain medication regimen was changed. Her fentanyl patch was discontinued, and she was discharged on hydromorphone for pain management. She was then re-admitted to the medical service for the management of withdrawal symptoms and was being followed by the acute pain service for adjustment of pain medications. Patient admitted to staff that she had previously tried to overdose on hydromorphone, in the setting of ongoing chronic pain and uncontrolled anxiety. The psychiatry consultation liaison service was consulted for evaluation of possible suicide attempt and for medical management of depression and anxiety.

Upon initial interview, the patient denied suicidal ideation, intent, and plan, but complained of ongoing anxiety symptoms and appeared acutely anxious. Her level of anxiety was interfering with her social functioning, as well as her ability to comply with medical treatment. She had been unable to work and was no longer able to travel because of worsening physical and mental health. The high level of anxiety led to hopelessness and fear that her condition would not improve, and concern that she would continue to require lengthy inpatient hospitalizations.

The patient’s past psychiatric history included diagnoses of unspecified depression and anxiety. She had multiple prior suicide attempts, cutting behaviors, and three prior psychiatric hospitalizations. Patient was not following with any outpatient psychiatrist prior to our evaluation. She reported multiple prior drug trials (durations and doses unknown) with adverse side effects, including paroxetine (suicidality), sertraline (suicidality), duloxetine (shakiness and tremors, poor impulse control, decreased sleep; discontinued due to concern for hypomania), amitriptyline (no improvement in symptoms), quetiapine (worsened anxiety), lorazepam, diazepam (both caused worsening paranoia), and gabapentin (vomiting, shakiness, and did not help improve pain symptoms). The only medications that the patient reported had been helpful in reducing anxiety were haloperidol and midazolam. Initially, venlafaxine 37.5 mg (crushed and administered through jejunostomy tube) was trialed, however, it caused worsening anxiety and gastrointestinal distress. Patient was then trialed on monotherapy of risperidone 0.5 mg oral at bedtime, with subsequent improvement in anxiety symptoms. She reported lessened anxiety and overall improved mood, and she was satisfied with the improvement in her symptoms. She became less preoccupied with somatic complaints and her lessened anxiety led to improvement in the quality of sleep. She was discharged home with this prescription and arranged with outpatient psychiatric follow up.

## Discussion

The chief complaint of patients with EDS frequently involves high levels of pain, which can lead to difficulties with diagnosis due to its subjective quality. Years of ongoing, and often untreated, suffering prior to a formal diagnosis can lead to patterns of negative thinking including catastrophizing, filtering, personalizing, generalizing, and polarizing [4]. These cognitive distortions can be precipitants for anxiety-related symptoms. The delay in diagnosis and uncertainty of the source of underlying pain, which are due in part to both society and physician’s unfamiliarity with EDS, contributes to an increased level of anxiety. The anxiety-related symptoms are often unrecognized or not prioritized because of the medical complexity of these patients. These patients often report feeling marginalized, alone, strange, or misunderstood [4]. These feelings can lead to psychological distress and these patients would benefit from earlier referral to psychologist and psychiatrists for support.

A cross-sectional study that assessed 80 patients with hypermobile EDS in France divided patients into two groups based on self-report: low and high anxiety levels. 51.2% of participants were categorized as experiencing a high level of anxiety [1]. In 2008, a postal survey was used to analyze 250 patients with EDS in Sweden, using the Hospital Anxiety and Depression Scale (HADS) and Short Form Health Survey (SF-36) scales. On the HADS, 74.8% scored high on anxiety, and 22.4% scored high on depression. The EDS group scored significantly lower on the SF-36 scale, indicating that patients with EDS perceived their physical and mental quality of health to be lower than the general population [7]. The high prevalence of anxiety in patients with EDS, as well as their perceived low quality of mental health, indicate the need for a multidisciplinary approach to these patients including psychiatric support and management.

Delays in diagnosis and treatment of anxiety can lead to poorer social functioning, as well as pain catastrophizing and sensory amplification [3]. For example, patients with EDS who were found to be highly anxious scored significantly higher in pain catastrophizing and somatosensory amplification when compared to their non-anxious counterparts [1]. These patients were also found to score lower in social functioning when compared to similar patients without anxiety [1]. Similar to other reports in the literature, this patient had uncontrolled anxiety, but was unable to tolerate the many side effects associated with different psychotropics. Her anxiety interfered with her ability to care for her children, maintain social relationships, and be an active member of the community. Therefore, her overall EDS prognosis can be expected to be worse than her non-anxious counterparts due to her untreated status. Patients who present similarly should prompt clinicians to treat the anxiety disorders aggressively due to the large potential for improvement of not only their anxiety symptoms, but also their psychosocial outcomes. 

Patients with EDS often present to major academic or community hospitals where psychiatry is often consulted for the management of comorbid psychiatric symptoms. The challenge for delivering proper care to this patient population is that this patient population has several other comorbidities that may cause untoward physical side effects from most antidepressants. Furthermore, while there is sufficient literature on the association between EDS and anxiety disorders, guidelines on the treatment of anxiety disorders in these patients continue to be lacking. There are many standard treatment options for anxiety disorders, but as seen in our patient, they are often inadequate or poorly tolerated due to extensive side effects. SSRIs are considered first-line treatment, though they should be used with caution in patients prescribed tramadol and codeine due to the risk of serotonin syndrome. TCAs, such as imipramine and clomipramine, can be used for the management of anxiety and depression but may cause orthostatic hypotension. MAOIs, which are no longer prescribed as frequently as they were in the past, can also cause orthostatic hypotension. Benzodiazepines can be useful in managing acute anxiety symptoms, and may decrease somatic sensitivity and secondary pain, but there is a risk of dependence, withdrawal, and cognitive changes [5].

To our knowledge, there are no current studies discussing the use of risperidone for the management of anxiety symptoms in patients with EDS. This patient provided significant challenges for treatment due to her complex history and inability to tolerate many of the previously mentioned medications. Her prior positive experience with haloperidol prompted our attempt to manage her anxiety with a different antipsychotic. Risperidone was chosen due to its less severe side effect profile. Risperidone is known to have side effects, including EPS and metabolic syndrome, however, using low doses can minimize these risks [8].

Studies have investigated the anxiolytic properties of risperidone, both as monotherapy and when used to augment other psychotropic drugs. In a randomized control trial that compared risperidone to paroxetine for the use of panic attacks, risperidone was found to work just as well as paroxetine. It was also noted to have a quicker clinical response even when used at low doses averaging 0.53 mg/day [9]. In studies done using rat models, Risperidone use at low doses has been shown to enhance antidepressant and anxiolytic effects of antidepressants [8]. The mechanism behind the success of risperidone in the treatment of various anxiety disorders may be related to its receptor profile. Risperidone more potently binds to serotonin 5-HT2A receptors than to dopamine D2 receptors, and also has a low affinity to histaminergic H1 receptors. The dual action of risperidone, as it functions as both a serotonergic and dopaminergic antagonist, may contribute to its anxiolytic effects [9].

Risperidone was successful in alleviating our patient’s anxiety symptoms. When her anxiety symptoms improved, she was better able to participate in treatment planning with her primary team, which resulted in an improved relationship with her doctors and better adherence to the recommended treatment options, as well as improvement in her level of psychosocial functioning. Just as management of psychiatric symptoms in patients with diabetes or cardiac dysfunction is crucial, the treatment of patients with EDS should also be explored further to give these patients a chance at a better quality of life. Anxiety disorders are associated with several medical disorders however the more prevalent diseases end up being studied and spoken of in publications more often. This article emphasizes the importance of treating medical disorders that have a high association with anxiety disorders such as EDS. If we can have a means of treating debilitating anxiety in a patient with EDS, then this should be studied and spoken about because of the great physical burden these patients already face. If we can broaden the realm of psychiatric treatment to very specific disorders such as EDS, then this can further elucidate the importance of addressing mental health along with physical illness, a challenge that the field of psychiatry still faces today. The successful use of risperidone in our patient with EDS suggests that it may be beneficial in treatment-resistant anxiety in other patients suffering from EDS. However, further research should be conducted to investigate the mechanism of this effect.

## Conclusions

Both literature and clinical experience indicate that psychiatric disorders, including anxiety disorders, are comorbid with EDS. However, there is minimal literature published regarding the management of treatment-resistant anxiety in this patient population. The above case describes the successful use of risperidone to reduce anxiety in a patient with EDS, indicating that risperidone may be helpful in managing anxiety disorders in these patients. The mechanism behind the improvement in anxiety symptoms may be due to risperidone’s dual antagonism of both serotonergic and dopaminergic receptors. We believe that further research into risperidone, as well as other atypical antipsychotics as a treatment option in this patient population is warranted. This can help attain the goal of enhanced quality of life for patients with EDS who already suffer a great deal of physical burden from their illness.
